# Condensin complexes: understanding loop extrusion one conformational change at a time

**DOI:** 10.1042/BST20200241

**Published:** 2020-10-02

**Authors:** Erin E. Cutts, Alessandro Vannini

**Affiliations:** 1 Division of Structural Biology, The Institute of Cancer Research, London SW7 3RP, U.K.; 2 Fondazione Human Technopole, Structural Biology Research Centre, 20157 Milan, Italy

**Keywords:** chromosomes, condensin, DNA binding, single-molecule, SMC, structural biology

## Abstract

Condensin and cohesin, both members of the structural maintenance of chromosome (SMC) family, contribute to the regulation and structure of chromatin. Recent work has shown both condensin and cohesin extrude DNA loops and most likely work via a conserved mechanism. This review focuses on condensin complexes, highlighting recent *in vitro* work characterising DNA loop formation and protein structure. We discuss similarities between condensin and cohesin complexes to derive a possible mechanistic model, as well as discuss differences that exist between the different condensin isoforms found in higher eukaryotes.

## Introduction

**Figure 1.**
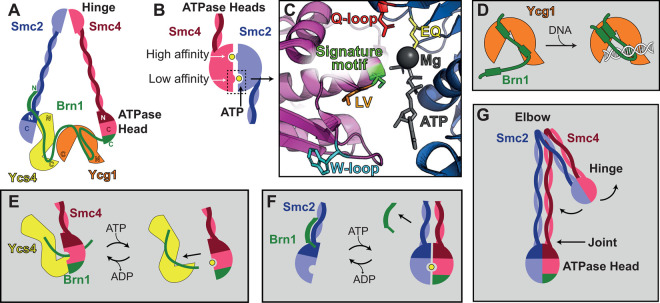
Condensin conformational changes. (**A**) Schematic of the subunits that make up *S. cerevisiae* condensin, indicating the hinge dimerisation domain, and ATPase head domains. (**B**) Schematic of ATP bound ATPase head domain, indicating high and low affinity ATPase sites in Smc4 and Smc2, respectively. (**C**) An atomic model of engaged Smc2 and 4 ATPase heads, created by superimposition of *C. thermophilum* Smc2 and 4 crystal structures (6QJ0 and 6QJ2) [43] with *C. thermophilum*/*S. cerevisiae* chimeric engaged Smc1/3 heads (6QPW) [50]. Commonly used mutations to prevent ATP binding (Q-loop, red), prevent head dimerisation (signature motif, green) and slow ATP hydrolysis (EQ, yellow) are indicated, as well as W-loop (cyan) important for binding Ycs4 and the LV mutation (orange) which affects ATP hydrolysis if present in Smc2 and Z-loop formation if present in Smc4. (**D**) Brn1 creates a safety belt, locking DNA to the Ycg1 HAWK subunit. (**E**) Ycs4/Brn1 binds Smc4/Brn1, but ATP interferes with this interaction, creating a possible ATP dependent cycle. (**F**) Brn1 N-terminus binding to Smc2 is lost in the presence of ATP dependent head dimerisation, creating another possible ATP dependent cycle. (**G**) SMC dimers are able to bend at the elbow region such that the hinge moves towards the ATPase heads. SMC coiled-coils also have some flexibility at the joint region, near the ATPases heads.

Organising DNA throughout the cell cycle to ensure correct gene expression, DNA replication, and chromosome division is a remarkable feat. Two structural maintenance of chromosomes complexes (SMCs), condensin and cohesin, contribute greatly to this process [1]. Accordingly, misregulation or mutation of either condensin or cohesin is associated with human disease, such as cancer and developmental disorders [2–5]. Condensin compacts DNA into chromosomes during mitosis and is also thought to play a role in genome architecture and transcriptional regulation [6–11]. Cohesin holds sister chromatids together and, along with CTCF, contributes to the formation of topologically associating DNA domains (TADs) [4,12,13]. Condensin and cohesin complexes share a similar molecular architecture (Figure 1A) [14,15] which, for simplicity, will be introduced using *S. cerevisiae* nomenclature (a list of homologues can be found in Table 1). Both complexes contain a pair of structural maintenance of chromosome (SMC) proteins, Smc2/4 and Smc1/3 for condensin and cohesin, respectively. SMC proteins are antiparallel coiled-coil proteins that hetero-dimerise via a hinge domain (Figure 1A). At the opposite end to the hinge is a split ATPase domain, harbouring two distinct ATP binding sites. While the first SMC head domain of the heterodimer binds ATP via a pocket containing the Walker A/B motifs, the second SMC head sandwiches the ATP molecule and provides the signature motif required for ATP hydrolysis. Mutation of key residues can prevent ATP binding (Q-loop), head engagement (signature motif) or slow ATP hydrolysis (EQ) (Figure 1B,C) [16–18]. A Kleisin protein, Brn1 in condensin and Scc1 in cohesin, binds to the SMC proteins to create a tripartite ring [19,20]. The Kleisin is bound by two heat repeat proteins [21], referred to as HAWKs [22] (HEAT proteins Associated With Kleisins). In condensin, the HAWKs are Ycg1 and Ycs4. In cohesin, however, the HAWKs are more diversified; Scc3 is bound to the C-terminal middle section of Scc1, while Scc2 and Pds5 compete to bind the N-terminal middle section of Scc1 [23]. Compared with yeast, metazoans have evolved additional condensin and cohesin isoforms. Humans have two isoforms of condensin; I and II, while cohesin has additional isoforms of the SMC1, STAG and PDS5 subunits (Table 1).

**Table 1:** Equivalent subunits that make up non-meiotic condensin and cohesin complexes

Species	Name	SMC Subunits		Kleisin	HAWKS	
*S. cerevisiae*	Condensin	Smc2	Smc4	Brn1	Ycs4	Ycg1
*S. pombe*	Condensin	Cut14	Cut3	Cnd2	Cnd1	Cnd3
*H. sapiens*	Condensin I	SMC2	SMC4	CAP-H	CAP-D2	CAP-G
*H. sapiens*	Condensin II	SMC2	SMC4	CAP-H2	CAP-D3	CAP-G2
*S. cerevisiae*	Cohesin	Smc3	Smc1	Scc1	Scc2 or Pds5	Scc3
*S. pombe*	Cohesin	Psm3	Psm1	Rad21	Mis4 or Pds5	Psc3
*H. sapiens*	Cohesin	SMC3	SMC1a^1^	RAD21^1^	NIBPL or PDS5A/B	STAG1/2^1^

1 *H. sapiens* also have meiotic specific cohesin SMC, Kleisin and HAWK subunits, referred to as SMC1b, REC8 and STAG3, respectively.

## DNA compaction and loop-extrusion

**Figure 2.**
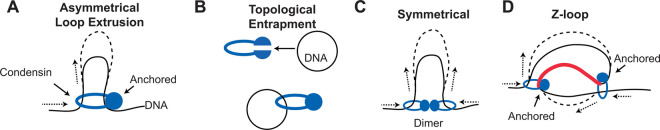
SMC loop extrusion. (**A**) Asymmetric loop extrusion, where a loop of DNA passes through a condensin ring anchored to DNA on one side. (**B**) Topological loading of SMC complexes, such as cohesin, is achieved by opening of the SMC-Kleisin ring to allow DNA to enter. (**C**) Symmetrical loop extrusion by a dimer of condensin complexes. For this to be achieved, one condensin complex extrudes DNA on each side, with neither condensin complex anchored to the DNA. (**D**) A condensin Z-loop, formed when two condensin complexes pass each other on DNA. The region of DNA in red has been compacted by both condensin complexes, hence the total amount of DNA compacted by a Z-loop is less than if two condensin complexes formed two separate loops.

DNA loop extrusion is a process whereby a loop of DNA is extruded through the SMC ring (Figure 2). Several recent studies using *in vitro* single-molecule approaches illustrate *S. cerevisiae, X. laevis* and *H. sapiens* condensin and cohesin complexes compact DNA and extrude DNA loops in an ATP dependent manner [17,24–31]. Cohesin activity requires the presence of the loader complex Scc2/NIPBL [29,30], while loop extrusion cannot be observed in the presence of PDS5A/B [28]. Both *H. sapiens* condensin and cohesin can compact DNA in the presence of nucleosomes, suggesting DNA bound proteins may not be obstacles to activity [27,29]. Loop-extrusion assays using cell lysates demonstrate that condensin and cohesin activity is cell cycle regulated; cohesin is responsible for the formation of the majority of loops in interphase, while condensin is responsible for loop establishment in mitotic extracts [31]. Additionally, single-molecule experiments using *S. cerevisiae* cohesin observe that two separate pieces of DNA can be tethered together by cohesin, which is thought to occur via topological entrapment of DNA, where the SMC ring opens such that DNA enters (Figure 2B). However, while DNA tethering is not observed in similar assays performed with condensin, condensin is able to translocate along one piece of DNA while transporting a separate piece [24,27,30]. There is also evidence that multiple pentamers can work together, with oligomerisation of *S. cerevisiae* condensin increasing DNA compaction activity in magnetic tweezer experiments [32] and single-molecule fluorescence studies suggest dimers of *H. sapiens* condensin and cohesin extrude symmetrical DNA loops (Figure 2C) [27,29]. In contrast, single-molecule fluorescence assays have shown *S. cerevisiae* condensin complexes predominantly extrude loops asymmetrically (Figure 2A), and when multiple condensin complexes collide on DNA they create higher-order Z loops, in which three double-stranded DNA helices align in parallel with one condensin at each edge (Figure 2D) [33]. Although single-molecule studies have shown loop-extrusion in real-time, the mechanistic role of each subunit and ATP remains largely unknown, however, recent structural studies have begun to address this.

## Brn1 buckles DNA to Ycg1

The crystal structure of the *S. cerevisiae* condensin HAWK, Ycg1 and Kleisin, Brn1 in complex with DNA suggest that Brn1 folds over and interact with DNA via a patch of conserved positively charged residues [34]. This structure appears to be conserved from yeast to human condensin I, with the *H. sapiens* Ycg1 homologue, CAP-G, having the same fold [35]. Mutation of positive-patch residues in the Kleisins Brn1 or CAP-H in yeast and human cells, respectively, results in loss of condensin chromosomal localisation [34]. Condensin complexes that lack Ycg1 or harbour mutations in the Brn1 positive patch are unable to compact DNA in magnetic tweezer experiments [17], resulting in a model where Brn1 acts as a ‘safety belt', tethering DNA to Ycg1 (Figure 1D). This is thought to enforce asymmetric loop extrusion (Figure 2A), however, translocation has also been observed for *S. cerevisiae* condensin [24] and symmetrical loop extrusion (Figure 2C) has been observed in *H. sapiens* condensin complexes [27]. Possible mechanisms we can propose to explain this are that DNA can slide beneath the latched safety-belt or that the safety belt opens and closes during loop extrusion. Chromosome assembly assays performed by Kinoshita et al. show that condensin I complexes lacking the CAP-G subunit can localise to DNA and form chromosomes, suggesting human tetrameric condensin complexes may maintain some functionality. However, these chromosomes are both longer and thinner than those formed by pentameric complexes, suggesting CAP-G plays a key role in shaping chromosomes [36]. Additionally, Brn1 may help regulate Ycg1 function by increasing Ycg1 rigidity and preventing Ycg1 oligomerisation and aggregation [37].

The Ycg1/Brn1/DNA structure bears striking similarity with the *S. cerevisiae* cohesin subunits Scc3 and Scc1 bound to DNA, in which the Kleisin Scc1 also contributes to the DNA binding affinity [38]. However, contrary to what is observed in condensin complexes, there is little evidence that the cohesin Kleisin subunit folds over DNA. In single-molecule experiments, while condensin remains bound to DNA after ATP is washed out [17], loss of ATP or NIPBL/MAU2 results in human cohesin loop release [28], suggesting that cohesin is less stably tethered to DNA. This could be explained by the discovery of the ‘gripping state' where Scc2/NIPBL within a cohesin complex firmly grips DNA in the presence of ATP [39–41]. In the case of human cohesin, lower DNA association stability might be compensated for by additional DNA binding factors, such as CTCF, which binds directly at the Scc3/Scc1 interface [42].

## Ycs4 binding to Smc4 is an ATP sensitive switch

The recent crystal structure of the other condensin HAWK, Ycs4, from *C. thermophilum* suggests it plays an essential role in regulating the Smc4 ATPase domain [43]. The co-crystal structure of Ycs4/Brn1 bound to the Smc4 ATPase, suggests that Ycs4/Brn1 binds to Smc4 via conserved sites on Ycs4 and Smc4, referred to as the KG-loop and W-loop, respectively. Comparison of this structure with the *S. cerevisiae* engaged Smc1 ATPase domain homodimer structure (PDB: 1W1W [44]) suggests that Smc4 can not bind to Ycs4 at the same time as Smc2. Furthermore, the addition of ATP inhibits Ycs4/Smc4 complex formation and mutation of either the KG-loop or the W-loop reduces the ATPase rate, suggesting that Ycs4/Brn1 can regulate the ATP binding cycle (Figure 1E). Both the KG and W-loops are conserved in humans and mutation of homologous residues in human CAP-D2 impairs loading onto chromosomes, suggesting this layer of regulation is likely to be conserved in humans [43]. Sequence analysis by Hassler et al. suggests that the W-loop could also be present in the cohesin subunit Smc1, and if we examine crosslinking data from Bürmann et al. [45] we find the W-loop of Smc1 crosslinks to Scc2 (Smc1:1124-Scc2:1193), suggesting that in the absence of ATP, these might interact similarly to Smc4 and Ycs4. Furthermore, Scc2 plays a role in regulating the ATP hydrolysis rate and is required for maximal ATPase activity of cohesin [46]. However, the cryo-EM structures of *H. sapiens* and *S. pombe* cohesin in an ATP-bound state show that Scc2 homologous subunit contacts SMC1 even in the presence of engaged heads [39,40]. This could reflect differences between the cohesin and condensin complexes, or simply a difference in how subunits behave in the presence or absence of DNA.

## The role of ATP

Recent work by Hassler et al. has provided mechanistic insights into condensin architecture and regulation by solving the crystal structures of the ATPase domains of Smc2 and Smc4 from *C. thermophilum*. Comparison of the Smc2 structure to ATPγS bound dimers of cohesin Smc1 heads suggests key differences in the ATP binding site of Smc2, resulting in Smc2 having a low binding affinity for ATP. In contrast, Smc4 displays high affinity for ATP, and ATP binding to this site alone is sufficient to promote ATPase head dimerisation, which in turn stimulates binding of ATP to Smc2 (Figure 1B) [43]. Consistent with this finding, extensive screening of Smc4 ATPase site mutants identified specific mutations that reduced condensin activity, while leaving the Smc2 ATPase domain unperturbed [47]. Interestingly, Elbatsh et al. found that mutation of a conserved leucine residue to valine near the signature loop (referred to in the text as LV, Figure 1C) of SMC2 or SMC4 results in markedly different phenotypes in human cells. Smc2-LV reduces the ATP hydrolysis rate of *S. cerevisiae* condensin, and SMC2-LV results in fuzzy, poorly condensed chromosomes, while mutation of SMC4-LV did not significantly affect ATPase rate and results in highly condensed chromosomes. This is consistent with Smc4 and Smc2 having high and low-affinity ATP binding sites, respectively. Both mutations in *S. cerevisiae* condensin were able to compact DNA and extrude DNA loops, with Smc2-LV mutant having slower DNA compaction and loop extrusion rates, in line with its reduction in ATPase rate. Smc4-LV however, was able to compact DNA faster, while still displaying the same loop extrusion rate. Further investigation found that Smc4-LV could not form Z-loops, and where two independent DNA loops result in more DNA compaction than two condensin complexes creating a Z-loop, this could account for faster DNA compaction while maintaining the same loop extrusion rate (Figure 2D). Similar results were observed in human cells, as Hi-C data suggests that SMC4-LV creates larger DNA loops than wild-type [48]. The mechanism behind how two condensin complexes interact to form Z-loops is not known, but if we examine the location of the Smc4-LV mutation, we see it is proximal to the W-loop of Smc4 found to bind Ycs4 (Figure 1C). Hence, we speculate that Smc4-LV might alter the Smc4/Ycs4 interaction in a way that prevents two condensin complexes passing each other on DNA, whether this is within one condensin pentamer or possible interactions between pentamers.

Hassler et al. also presents an NMR structure of a fusion made from the N-terminus of Brn1 with two helices of the Smc2 coiled-coil proximal to the ATPase domain. Overlay of the Brn1/Smc2 fusion structure with the crystal structure of *C. thermophilum* Smc2, suggests that Brn1 binding results in a conformational change in the Smc2 coiled-coil helices. Using a pentameric complex with a TEV protease cleavage site on Brn1 between the Smc2 and Ycs4 binding sites, the authors proceed to show that binding of ATP results in loss of the N-terminal fragment of Brn1, while mutations of Smc2/4 preventing ATP binding or reducing head engagement retain Brn1. This suggests a mechanism whereby ATP binding and head engagement opens the condensin ring by releasing the N-terminus of Brn1 (Figure 1F) [43].

Similarly in cohesin, the N-terminal region of the Kleisin Scc1 releases upon addition of ATP or ATPyS [49,50]. This is associated with cohesin unloading in the presence of Pds5 and Wapl [49,51–53] where a fusion of the Smc3 C-terminus to the Scc1 N-terminus reduces unloading [54]. However, this has recently been implicated in *S. pombe* cohesin loading, where FRET experiments detect N-terminal Kleisin release upon ATP binding and an increase in N-terminal Kleisin occupancy in the presence of Mis4, DNA and non-hydrolysable ATP [40]. Release of the Kleisin is not thought to be required for loop extrusion activity, as a trimeric fusion of SMC3–RAD21–SMC1a with crosslinked hinge domains are still able to extrude DNA loops [28]. However, cryo-EM structures of *H. sapiens* and *S. pombe* cohesin with engaged ATPase heads clearly show the N-terminus of the Kleisin, Rad21 binding to SMC3. This suggests either a conformational change of coiled-coils results in the temporary release of Rad21 or that the presence of DNA and/or the loader NIBPL/Mis4 prevents release or contribute to rebinding of Rad21 to SMC3/Psc3 [39,40]. Based on similarity, the release of N-terminal Brn1 could contribute to loss of condensin DNA loops, and if ATP binding to Smc2 promotes release, it might explain why condensin has evolved such that Smc2 has low ATP binding affinity. However, unlike cohesin, there is little evidence that condensin has release factors and the proposed mechanisms for DNA decompactions include post-translational modifications of condensin subunits, alteration at the protein level of condensin subunits and degradation [55–58].

## SMC coiled-coil conformation

A striking feature of recent *H. sapiens* and *S. cerevisiae* condensin structural work is a bend in the coiled coils ∼15 nm from the hinge [27,59]. This bend, referred to as the ‘elbow', has also been observed in EM analysis and crosslinking data of *S. cerevisiae* and *S. pombe* cohesin and *E. coli* MukBEF SMC-Kleisin, and results in the hinge bending to contact the SMC arms near the ATPase domains [40,60]. In cohesin, crosslinking and FRET based studies support the hypothesis that the hinge may fold to contact *S. pombe* Pds5, Psc3 or Mis4 [49,61,62] and cryo-EM structures of *H. sapiens, S. cerevisiae* and *S. pombe* suggest that the hinge folds to contact the HAWKs [39–41]. Folding of the SMC arms is observed in recent AFM data of *S. cerevisiae* condensin, showing that the hinge can fold towards the ATPase domains, however, does so with the SMC arms open, transitioning from an open O to a B shaped conformation [63]. Hence, folding of the SMC hinge towards the globular region (Figure 1G) is a conserved feature in SMC complexes. As the SMC hinge domains have been found to bind DNA [64–68] and *S. cerevisiae* condensin and cohesin have been observed to interact with DNA via both their hinge and globular domains [41,63], folding is likely to have a key role in the loop extrusion mechanism.

## Holocomplex structure

While individual crystal structures have provided much needed molecular detail of subunit interfaces, full understanding of the mechanism requires structural information on the intact complex in different functional states. Recent work by Lee and Merkel et al. [59] have begun to address this gap in knowledge by determining cryo-EM structures of *S. cerevisiae* condensin in the presence and absence of ATP. Despite using pentameric complexes for much of their analysis, only one HAWK domain is visible in each structure, suggesting the HAWKs are highly dynamic. Two conformations were determined in the absence of ATP (Apo). In both conformations Ycs4 binds the Smc4 ATPase head, consistent with the Ycs4/Smc4 crystal structure presented by Hassler et al. [43], however the conformation of the ATPase heads with respect to each other differs. In one conformation the heads are close together, but not near enough to sandwich ATP, while in the other the ATPase heads are separated by ∼10 nm, with Ycs4 bridging the gap by binding Smc2. In the ATP bound structure, the ATPase heads are engaged and no density is visible for the N-terminal region of Brn1, again consistent with Hassler et al. [43]*.* This structure reveals that Ycg1 interacts with the Smc2 ATPase head. Mutation of this interaction region in yeast results in reduced cell viability, suggesting it could be physiologically relevant. Further experiments determined that binding of Ycs4 to Smc4 in the absence of ATP is mutually exclusive to Ycg1 binding to Smc2 in the presence of ATP. This suggests a domain ‘flip-flop' mechanism, where HAWKs flip in and out to allow loop extrusion. However, none of the recent condensin EM structures were determined in the presence of DNA, hence we can only speculate where DNA resides within the structure during ATP hydrolysis and loop extrusion.

**Figure 3.**
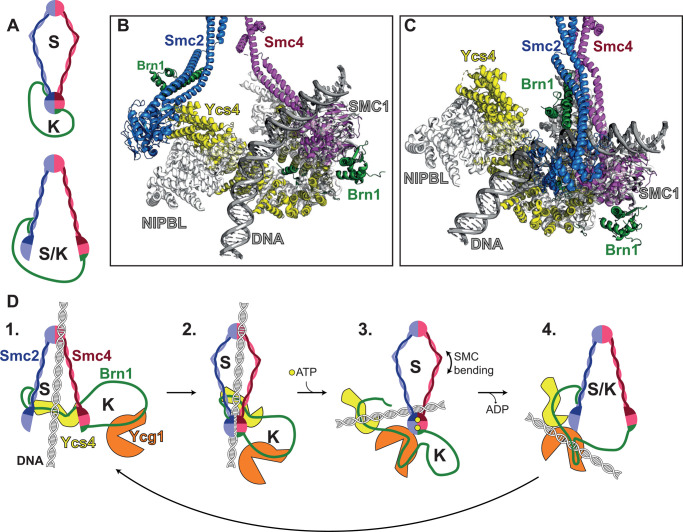
A potential mechanism for condensin DNA binding. (**A**) SMC complexes can create two distinct DNA entrapment compartments, the S-compartment (S), made by the SMCs and the K-compartment (K), made by the Kleisin. Opening of the SMC ATPase heads could result in fusion of these compartments (S/K). (**B**) Overlay of the *S. cerevisiae* bridged Apo condensin structure (coloured by domain, PDB: 6YVV) with NIPBL, SMC1 ATPase domain and DNA from the *H. sapiens* cohesin structure (shown in white/grey, PDB: 6WG3). This places DNA in contact with the top of the Smc4 ATPase domain and a region of Ycs4 with positive surface charge, discussed by Lee and Merkel et al. [59]. (**C**) Overlay of the *S. cerevisiae* Apo condensin structure (PDB: 6YVU) with NIPBL, SMC1 ATPase domain and DNA from the *H. sapiens* cohesin structure (PDB: 6WG3), suggesting the ATPases heads clamp around DNA after it is bound by Ycs4. (**D**) The proposed mechanism for condensin DNA binding during ATP turn over.

Current models of *S. cerevisiae* cohesin and *B. subtilis* Smc-ScpA [69,70] suggest SMC complexes have of two distinct entrapment compartments, referred to as the S compartment (as it is formed by the SMCs), and the K compartment (formed by Kleisin), which can fuse to create a larger S/K ring (Figure 3A). The investigation into K and S compartments in *S. cerevisiae* cohesin suggest Scc2 promotes ATP independent DNA entry into both the S and K compartments, but not the S/K ring, while Scc3 and ATP hydrolysis is required for DNA entry into the K-compartment [41]. Cryo-EM work in this study, and by others on *S. pombe* and *H. sapiens* cohesin, show that DNA is gripped by Scc2 on one side and trapped by engaged ATPase heads on the other [39–41], hence, DNA could gain entry to the S and K compartments by DNA binding to Scc2, before the ATPase heads close around it. Overlay of the *S. cerevisiae* Apo condensin structures with NIPBL/SMC1/DNA from *H. sapiens* cohesin structure [39], suggest condensin could bind DNA in a similar manner (Figure 3B,C). While no structural data exist for Ycs4 directly binding to DNA, several evidences suggest that Ycs4 play a central role in condensin binding to DNA: a positively charged patch defines the Ycs4 putative DNA binding surface [59], Ycs4 is able to efficiently bind DNA in gel shift assays [34], and the *H. sapiens* condensin I Ycs4 equivalent, CAP-D2 is essential for chromosome formation in chromosome assembly assays [36], suggesting that this is essential in condensin binding to DNA.

Based on condensin existing structural work, and taking into consideration a general functional homology with cohesin, we propose the following steps in the mechanism of condensin loop extrusion (Figure 3D):

1. DNA binds to Ycs4 in the condensin bridged state. The hinge could additionally contribute to DNA binding.
2. DNA binding promotes closure of the ATPase heads, resulting in DNA trapped within both the K and the S compartment.
3. Proximity of ATPase heads promote ATP binding, release of Ycs4 and binding of Ycg1 to the Smc2 ATPase heads. SMC coiled coils bending could contribute to the relocation of DNA within the S compartment.
4. ATPase heads open after ATP hydrolysis, creating a temporary S/K ring, enabling DNA to pass into the K compartment. Once DNA is in the K-compartment it can bind Ycg1 and the Brn1 safety belt can close. The cycle can then repeat when Ycs4 binds the Smc4 ATPase head, displacing Ycg1.

## Human condensin and chromosome structure

**Figure 4.**
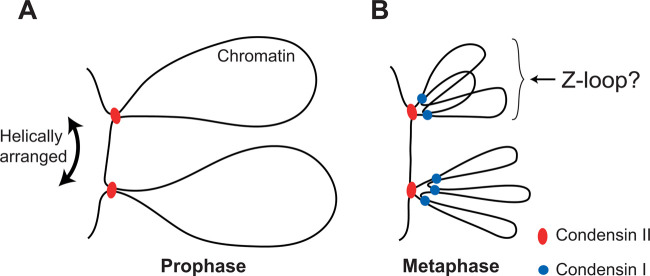
Chromosome formation by condensin loop extrusion. (**A**) At the start of mitosis in prophase, before nuclear envelope breakdown, condensin II is able to start loading on chromatin, resulting in the formation of large loops. (**B**) After nuclear envelope break down in metaphase, condensin I gains access to DNA, and forms multiple loops nested within the larger condensin II loops. Some condensin I loops could take the form of Z-loops.

*H. sapiens* condensin I and II are spatially separated during interphase; condensin II localises within the nucleus throughout the cell cycle, while condensin I is cytoplasmic during interphase and only gains access to the DNA after nuclear envelope break down, allowing condensin II to start loading before condensin I [71]. Depletion of either condensin I or II results in different chromosome morphology and genomic instability phenotypes. Condensin I depletion results in wider chromosomes and the formation of ultrafine bridges at anaphase, while condensin II depletion results in elongated, curly chromosomes and larger chromatin bridges [7,48,72–74]. Condensin I and II are also present on chromosomes in different ratios throughout mitosis, starting at roughly 2 times more condensin I than condensin II in prophase, going up to ∼6 times more in anaphase [75]. This ratio shapes chromosomes, where altering the ratio to 1 : 1 causes chromosomes to become shorter and thicker [76]. Despite condensin I being more abundant, condensin II seems to contribute more to the rigidity of chromosomes. Whole chromosome stretching assays indicate that condensin II-depleted chromosomes have a much larger decrease in elastic modulus than condensin I-depleted chromosomes [77]. Super-resolution microscopy has shown that both condensin I and II localise to an axis formed at the centre of chromosomes, with condensin II localising at the core of the axis, surrounded by condensin I. This work also suggested that in prometaphase condensin II forms loops ∼450 kb in size, while condensin I forms loops ∼90 kb in size [75]. In agreement with this work, *in silico* chromatin modelling and analysis of Hi-C data from DT-40 chicken cells where condensin I or II was rapidly depleted by auxin-inducible degradation of CAP-H or CAP-H2, respectively, suggest that in prometaphase condensin II promoted the formation of ∼400 kbp loops, while condensin I forms ∼80 kbp loops. Furthermore, this work suggests that condensin I and II loops form a helical axis at the core of chromosomes with ∼12 Mbp per helical turn, which results from the combined activity of condensin I and II. Condensin I and II alone form only narrow or wide helical axes, respectively, creating distinct diagonal stripes in the Hi-C plot [7]. Collectively, these studies suggest a model for mitotic chromosome formation, where first condensin II makes large DNA loops (Figure 4A), followed by condensin I making multiple smaller loops nested within the larger condensin II loops (Figure 4B). As Elbatsh et al. [48] demonstrated, mutations that prevent Z-loop formation cause a hyper condensed phenotype in human HAP1 cells deficient in condensin II, it is conceivable that some condensin I loops could take the form of Z-loops, and we speculate how Z-loops might contribute to chromosome structure (Figure 4B).

Altogether, these studies suggest the activities of condensin I and II differ *in vivo*. In agreement with this notion, purified recombinant condensin I and II also display different activity. Condensin II binds to DNA with higher affinity and compacts DNA with slower velocity than condensin I, despite a similar bulk ATPase activity [27]. Whether the intrinsic different biophysical properties of condensin I and II are sufficient to explain the differences observed *in vivo* remain to be addressed. In fact, the actions of condensin I and II *in vivo* could also be further modulated and regulated by co-factors present in the cellular environment. There is an ever-increasing list of condensin I or II specific co-factors including but not limited to, the chromo kinesin KIF4A, telomere-associated TRF1 and TANK1, cell cycle factors RB1, pRB and Plk1, chromatin modeller component, Arid1a, the transcription factor, TFIIIC and DNA damage response factor, MCPH1 [78–87]. The role of condensin I and II binding partners in the structural organisation of the genome is largely unknown.

## Future directions

There is still much work to be done in order to fully understand how condensin complexes work in the cellular environment. However, recent work has shown that throughout evolution, condensin complexes, as well as cohesin, can extrude DNA loops, a fundamental function which has remained conserved throughout evolution. Individual conformational changes that subunits of condensin undergo in the presence of ATP and DNA have started to be identified, but the full range of steps elucidating the locations of DNA binding surfaces throughout the ATPase cycle still need to be determined. While both condensin and cohesin can compact nucleosome-bound DNA, further work is required to better understand how these complexes works on chromatin. Recent work also poses a number of new questions. Does condensin (and cohesin) work symmetrically as dimers, asymmetrically as monomers or both? What is the mechanism underlying the formation of Z-loops? Do human condensin complexes and cohesin form Z-loops?

Although many questions remain, the recent advances described here illustrate the strength of integrating complementary approaches, such as *in vitro* biochemistry, Cryo-EM, single-molecule experiments and Hi-C methods. These results form a strong foundation to help build a detailed understanding of how condensin complexes function to organise chromatin in cells.

## Perspectives

- *Importance of the field:* Condensin complexes are essential for organising DNA into chromosomes during mitosis, and alterations in condensin function are associated with genome instability and cancer. Hence, structural and mechanistic understanding of condensin function is crucial for understanding its role in human disease.
- *Current thinking:* Condensin complexes extrude DNA loops using a mechanism that involves multiple inter-subunit interactions that undergo conformational changes in a DNA or/and ATP dependent manner.
- *Future directions:* High resolution Cryo-EM structures of condensin complexes bound to DNA throughout the ATPase cycle are needed to provide insight into the holoenzyme mechanism. Building on previous results by adding co-factors and chromatin will further enhance our understanding of how condensin works in cells and contributes to human disease.

## Abbreviations

AFM: atomic force microscopy
EM: electron microscopy
HAWK: HEAT proteins associated with Kleisins
SMC: structural maintenance of chromosome
